# Systems Biology-Derived Discoveries of Intrinsic Clocks

**DOI:** 10.3389/fneur.2017.00025

**Published:** 2017-02-06

**Authors:** Arthur Millius, Hiroki R. Ueda

**Affiliations:** ^1^Laboratory for Synthetic Biology, RIKEN Quantitative Biology Center, Suita, Osaka, Japan; ^2^Department of Systems Pharmacology, Graduate School of Medicine, The University of Tokyo, Tokyo, Japan

**Keywords:** systems biology, models, theory, RNA sequencing, neurophysiology, circadian rhythm, ribosome profiling

## Abstract

A systems approach to studying biology uses a variety of mathematical, computational, and engineering tools to holistically understand and model properties of cells, tissues, and organisms. Building from early biochemical, genetic, and physiological studies, systems biology became established through the development of genome-wide methods, high-throughput procedures, modern computational processing power, and bioinformatics. Here, we highlight a variety of systems approaches to the study of biological rhythms that occur with a 24-h period—circadian rhythms. We review how systems methods have helped to elucidate complex behaviors of the circadian clock including temperature compensation, rhythmicity, and robustness. Finally, we explain the contribution of systems biology to the transcription–translation feedback loop and posttranslational oscillator models of circadian rhythms and describe new technologies and “–omics” approaches to understand circadian timekeeping and neurophysiology.

## Systems Biology—A Brief History

In contrast to a reductionist approach, systems biology emphasizes the interaction of components rather than the components themselves: to see the forest for the trees. This holistic approach is not a modern idea, but can be traced as far back as the Greek Aristotle “…the totality is not, as it were, a mere heap, but the whole is something besides the parts…” In the modern era, Karl Ludwig von Bertalanffy is generally credited as one of the founders of general systems theory with his model of individual cell growth in the early 20th century (1). Later, the Dutch physicist Balthasar van der Pol working with electric circuits developed his eponymous equation to describe relaxation oscillations (2), which was used for theoretical models of neuronal systems (3, 4). In the 1950s, Alan Hodgkin and Andrew Huxley described the first mathematical model of an action potential propagating along a neuron, which famously predicted the existence of ion channels before their experimental discovery (5), and Alan Turing proposed a reaction–diffusion system in “The Chemical Basis of Morphogenesis” to explain how an initially homogenous system—the embryo—forms patterns through the action of morphogens (6).

These early systems models of cellular behavior were overshadowed by the excitement of the molecular biology revolution. Geneticists and biochemists learned to devise assays to measure the impact of single genes and single enzymes. In the 1970s, Ronald Konopka in Seymour Benzer’s lab used chemical mutagenesis to screen fruit flies for defects in their rhythmic emergence from the pupae state. He discovered three alleles of the Period gene, which is one of the earliest examples of a gene determining behavior in an organism (7). For the next 30 years, circadian biologists mostly pursued reductionist approaches similar to Konopka’s strategy to examine circadian behaviors in different organisms by knocking out single genes or isolating individual tissues.

**Figure 1:**
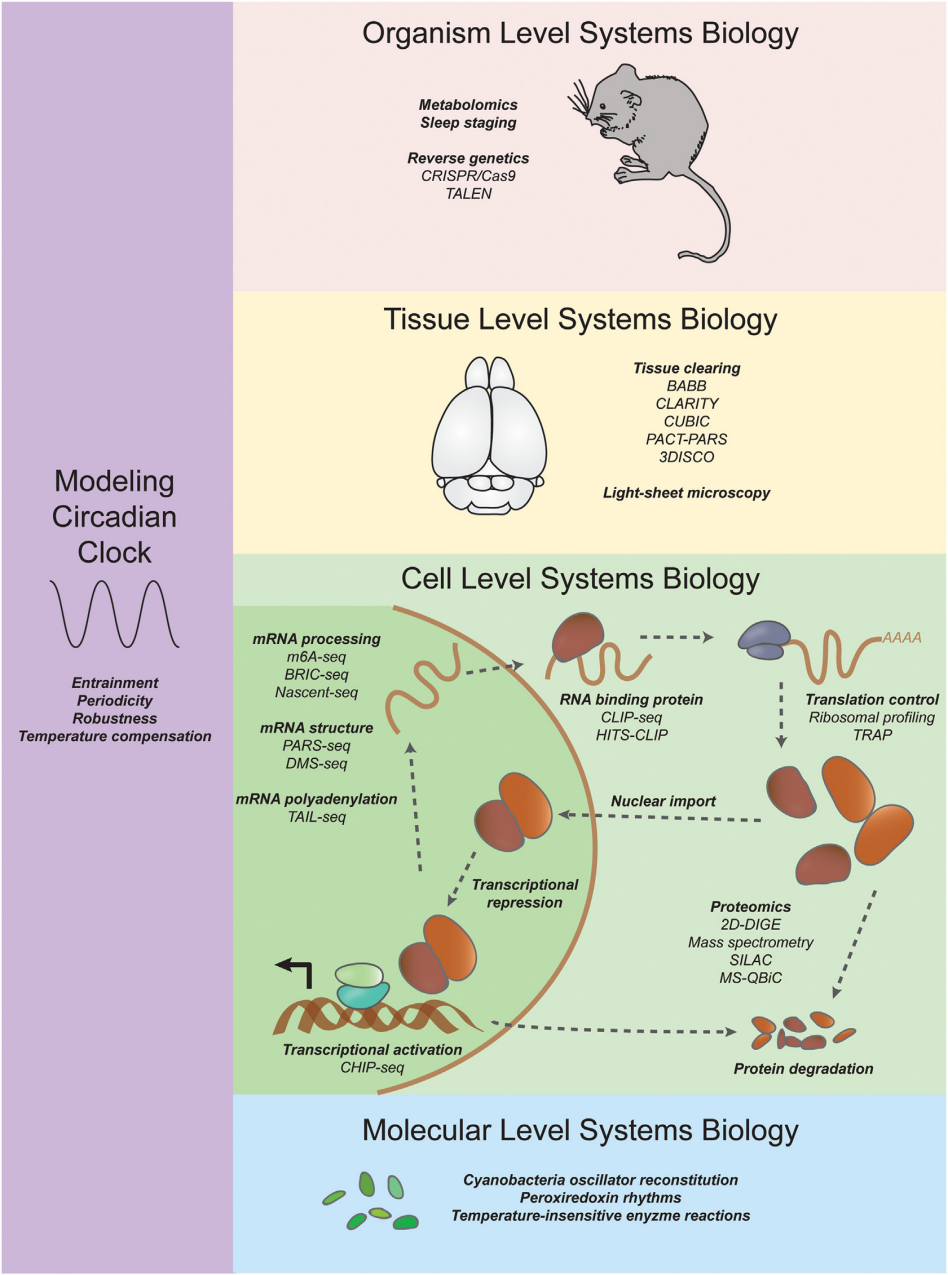
**Systems approaches to studying circadian rhythms**. On an organism level, researchers are using CRISPR/Cas9 and TALEN coupled with new sleep staging techniques to uncover mutations in genes that increase or decrease sleep. On a tissue level, new tissue clearing techniques such as CLARITY and CUBIC are enabling researchers to investigate the neuroanatomical basis of behavior (see Systems Neurophysiology). On a cell level, systems transcriptomics experiments have revealed not only rhythmic mRNA levels through microarrays and RNA sequencing but also other molecular details such as chromatin state, mRNA structure and modification, ribosome binding, and rhythmic protein abundance (see Systems Transcriptomics, Systems Proteomics and Metabolomics, and Systems Approaches to Study Translation Regulation in Circadian Rhythms). On a molecular level, reconstitution of a cyanobacteria posttranslational oscillator and the discovery of transcription/translation independent peroxiredoxin rhythms have expanded our understanding of circadian oscillations (see Periodicity and the Rise of the Posttranslation Circadian Oscillator). Systems modelers have discovered insights into constraints and parameters necessary for unique features of the circadian clock such as entrainment, periodicity, robustness, and temperature compensation (see Modeling the Systems Properties of Circadian Rhythms).

The era of functional genomics and next-generation sequencing has begun to shift the balance back toward systems biology. In the following sections, we review the contributions of mathematical models, microarray technology, RNA sequencing, proteomics, and neurophysiological approaches to systematically dissect circadian behavior and uncover new modes of regulation (for an overview, see Figure 1).

## Modeling the Systems Properties of Circadian Rhythms

The circadian clock is an interconnected network—a network of small molecules and metabolites, a network of genes and proteins, and a network of cells, neurons, and tissues. At each level, the interacting network of components can create complex behaviors. These systems-level properties include three defining characteristics of circadian rhythms: (1) periodicity—rhythms are autonomous with a period that matches the daily 24-h rotation of the Earth, (2) entrainment—rhythms can be reset by environmental cues such as light, temperature, or food intake, and (3) temperature compensation—periodicity of rhythms persistent despite fluctuations over physiologically relevant temperature ranges.

Before genetics led to the identification of molecular components governing a transcription and translation feedback loop that underlies the mechanism of circadian oscillation in many organisms, theoretical studies sought to model how oscillation, periodicity, entrainment, and temperature compensation could arise. The first was Goodwin’s model of a molecular oscillator using negative feedback (8–10). Understanding the different types of behavior in networks have enabled mathematical biologists to make predictions about which biological processes affect circadian rhythm behavior such as period length and temperature compensation. For example, in a hypothetical biochemical network with negative feedback, there are necessary constraints on reaction rates for the generation of instability at steady state (11). Using this constraint and other ideas from signal processing in the Goodwin model for circadian oscillation, it could be shown that transcription and translation rate are not important for setting period length, but instead a critical feature is the degradation rate of the repressor (12). These studies highlight the fundamental contributions of systems modelers even without knowledge of the molecular network underpinning circadian rhythms.

Identification of the molecular components of circadian rhythms led to an explosion of models incorporating these proteins and functions. Goldbeter’s model used non-linearity of Hill-type equations in the Goodwin model when he reported the first model of circadian rhythms based on observations of PERIOD phosphorylation and degradation in *Drosophila* (13). Non-linearity in feedback repression could occur through cooperative binding of multiple repressors to a promoter or via repressive multisite phosphorylation of a transcriptional activator. Derivations of this type of model have been used to examine *Drosophila* (13–19), *Neurospora* (14, 16, 20, 21), and mammalian circadian rhythms (22–30). In the next subsections, we discuss how these and other models contributed to our understanding of the systems properties of circadian rhythms.

### Periodicity and Design of the Transcription–Translation Feedback Loop

The period of a biological rhythm is tied to the 24-h rotational movement of the Earth. Organisms across different domains of life evolved timing mechanisms called biological clocks to coordinate function and behavior to specific times of the day (31). Each day environmental cues such as light and temperature reset your biological clock in a process called entrainment (32). Food can also entrain biological rhythms by affecting clock machinery in the liver (33, 34). Entrainment allows us to recover from the jet lag inducing effects of airplane travel by either advancing or delaying the phase of the circadian clock. Response to external cues is not instantaneous—timekeeping of the circadian clock persists, which is why we feel jet lagged in the first place.

Flexibility in period length was apparent from the earliest studies of mutant organisms (7, 35, 36). Systematic screening of chemical libraries also revealed chemical compounds that could alter period length by targeting specific clock proteins (24, 37–44). Pharmacological and/or genetic perturbation could extend the range of periods in the fibroblast from 27 to 54 h (41) and suprachiasmatic nucleus (SCN) from 17 to 42 h (45). Investigating why some mutant organisms have short or long periods revealed the molecular mechanisms of circadian rhythms and researchers could begin to test models by designing and manipulating components in the circuit. They were perhaps inspired by synthetic bacteria genetic circuits that recapitulate transcriptional oscillations (46) and bistable switches (47). For circadian rhythms, mathematical modeling guided construction of a synthetic 26-h oscillator based on siRNA-based silencing of a tetracycline-dependent transactivator (48). Construction of a mammalian promoter/enhancer database allowed researchers to identify high-scoring or low-scoring cis-elements and validate high- or low-amplitude expression, respectively, in cells (49), which enabled synthetic reconstruction of different circadian phases in cells by mixing combinations of promoter elements (50, 51). Researchers have also implemented artificial photic input pathways to clock cells to investigate singularity behavior, in which the circadian clock is reset after perturbations of different strengths and timing (52). More recently, researchers have succeeded in replacing the endogenous repressor in mice with a tunable one (53) and artificially manipulating the molecular circuitry of pacemaker cells in the brain (54, 55) to alter period length. These synthetic biology reconstruction experiments probe the sufficiency of circadian networks to generate oscillations and oscillations of different periods as well as test ideas about how network components interact and function within cells.

### Periodicity and the Rise of the Posttranslation Circadian Oscillator

Scientists originally thought that a transcription–translation feedback network was required for 24-h rhythms. But then, a remarkable study was published. Working in cyanobacteria, Kondo and colleagues mixed a small number of cyanobacterial proteins KaiA, KaiB, and KaiC, and ATP in a test tube to produce rhythmic 24-h oscillations in KaiC protein phosphorylation (56). In a manner similar to simple chemical reaction–diffusion systems creating Turing patterns, 24-h periodicity could be established in the absence of a transcription–translation negative feedback loop architecture.

A few years later, it was discovered that an antioxidant enzyme called peroxiredoxin in cultured human red blood cells undergoes temperature-independent circadian cycles of hyperoxidation. Because red blood cells lack a nucleus and peroxiredoxin rhythms persist in the presence of transcription and translation inhibitors, these rhythms prove the existence of a non-transcriptional-based circadian oscillator in mammals (57) and was later found to be conserved in a wide range of species (58). In mice, rhythmic peroxiredoxin oxidation is thought to occur through hemoglobin-dependent H_2_O_2_ generation and proteasome degradation (59), but it remains unclear how rhythmic oxygen delivery occurs in isolated cells and how the rhythms of peroxiredoxin oxidation are temperature compensated. In the future, a more detailed understanding of the relationship between rhythmic peroxiredoxin oxidation and canonical circadian clocks is needed.

The reconstitution of a phosphorylation oscillator in cyanobacteria (56) prompted modelers and synthetic biologists to question what the minimal components are for a circadian oscillator. In cyanobacteria, biochemical studies have driven our understanding of the mechanism of the oscillator. KaiC was discovered to be both a kinase and a phosphatase (60–62). KaiC autophosphorylation is triggered by allosteric activation by KaiA (63, 64) and regulated through feedback inhibition by KaiB (60, 65). Importantly, a sequential ordering of phosphorylation at two sites on KaiC is necessary for oscillation (66, 67) and remarkably, when Kai protein complexes from different starting phases are mixed, the phosphorylation state of the population remains in synchrony (68).

Several models have been proposed to explain the mechanism of oscillation (69–71) and synchrony of the cyanobacteria oscillator on a population level (67, 72, 73). A central idea is that there is monomer shuffling between KaiC hexamers, which was proposed in mathematical models (72, 74) and by experiments from the Kondo laboratory (65, 68), and confirmed elsewhere by FRET experiments (75). Other models do not explicitly rely on monomer exchange for synchrony (67, 73), but rather synchrony arises as an emergent property of the system based on KaiA’s affinity for different phosphorylated forms of KaiC. Of course, concepts such as differential affinity and monomer exchange have been incorporated together into more sophisticated models of cyanobacteria rhythms (76, 77).

Studies in cyanobacteria provide a foundation to understand the requirements (ordered phosphorylation, synchrony, etc.) for a generic phosphorylation oscillator. Most models of non-circadian phosphorylation oscillators require additional mechanisms for rhythmicity such as protein synthesis and degradation (78) or allosteric feedback from substrate (79, 80). However, a theoretical study demonstrated that autonomous circadian oscillations are possible with a single substrate reversibly phosphorylated at only two sites (81) and suggested that a well-defined ordering of phosphorylation states and sequestering checkpoints for enzyme activity could be design principles for single-molecule oscillators for the circadian clock and potentially other cellular oscillators. The Jolley model (81) results in a substrate with four possible modification states similar to MAPK (82) and cyanobacteria models (67). While a general phosphorylation oscillator has not yet been built based on these models, the reconstruction of temporal (56) and spatial (83) oscillators from purified components provide inspiration for future work. Furthermore, the recently reported success in transplanting the circadian clock from cyanobacteria into the non-circadian bacterium *Escherichia coli* (84) implies some amount generality for the network and design principles upon which circadian rhythms lie.

### Temperature Compensation

Insensitivity to temperature was originally identified as an essential characteristic of biological time-measuring systems in bees, flies, and marine organisms (85–88) and references therein. In particular, it was postulated that temperature independence was the result of a temperature compensation mechanism involving the opposing effects of enzyme activities in response to changes in temperature (87). Researchers began to identify genetic mutants with defects in temperature compensation in *Neurospora* (89, 90) and *Drosophila* (91, 92). In flies, repressor dimerization was thought to be involved in temperature compensation because loss of the repressor’s dimerization domain caused the period to strongly depend on temperature (91). Researchers incorporated these ideas into models of circadian rhythms by suggesting that nuclear import of the repressor decreases with temperature and repressor dimerization increases with temperature (93, 94). Other models emphasized the importance of degradation of the repressor (95, 96) and other parameters needed for temperature compensation (97). The conceptual point of these models is that for circadian rhythms to be temperature compensated, some biochemical reactions accelerate circadian oscillations, while other biochemical reactions decelerate circadian oscillations. The balance model supposes that the former acceleration reactions are less sensitive to temperature, whereas the latter deceleration reactions are more sensitive to temperature. A molecular basis for this type of temperature compensation was proposed in plants (98) and also formulated mathematically as a balance equation (99) to explain how *Neurospora* repressor stability decreases with an increase in temperature (95, 100), which is ultimately caused by phosphorylation-dependent degradation from a kinase (101).

In 1968, Pittendrigh and colleagues argued against a balancing model in which temperature shortens a reaction in the first half of a circadian cycle while simultaneously lengthening a reaction in the second half of the cycle in their experiments with *Drosophila* (102). They used short light pulses to shift the phase of *Drosophila* pupae at different temperatures and showed that the period and wave form of the phase response curve changes only a little bit with temperature. They proposed a model where circadian output from a temperature-dependent oscillation is subjected to feedback inhibition from another temperature-dependent reaction (102, 103). These early studies suggested a model in which the enzymatic reactions that comprise the clock are temperature compensated. However, the idea of a temperature-compensated enzyme is counterintuitive because most chemical processes are temperature dependent. In cyanobacteria, the kinetic profile of the phosphorylation to dephosphorylation ratio is temperature compensated *in vitro* (56, 104). This was the first indication that temperature compensation could occur through the enzymes themselves as opposed to compensation that occurs through competing biochemical reactions.

The canonical transcription–translation feedback loop underlying circadian rhythms in eukaryotes may also be affected by temperature-insensitive enzymatic reactions. In eukaryotes, it was first discovered in mammals that the phosphorylation-dependent degradation rate of the repressor is temperature insensitive in cells, and temperature-insensitive phosphorylation is preserved *in vitro* (41). This suggests that temperature-insensitive enzymatic reactions can influence the circadian transcription–translation network. In addition to component-level temperature compensation (41), detailed examination of the degradation of the repressor revealed three distinct stages of degradation that depend on when during the circadian cycle protein translation is arrested (105). The authors in this study suggested that temperature-insensitive and -sensitive phosphorylation at different sites of the repressor are responsible for temperature compensation. In the future, it will be particularly interesting to uncover the mechanisms and structural basis of temperature compensation in these individual reactions and to synthetically engineer temperature compensation in circadian clocks similar to synthetically temperature-compensated genetic networks in bacteria (106).

### Robustness to Gene Dosage

Circadian rhythms are surprisingly robust to changes in gene dosage—there has been much discussion about why knockout of core clock genes only results in subtle period lengthening or shortening (107). There have been efforts to understand networks effects by systematically altering individual gene levels (108) or by globally altering transcription levels with drugs (109). Resistance to internal noise from the stochastic nature of biochemical networks in the cell is an essential property for a robust circadian clock network (110). Theoretical models suggested that intercellular coupling between individual oscillator cells is necessary for synchrony and noise resistance (111). Indeed, dissociated SCN neurons and isolated cells from tissues such as lung and liver are arrhythmic compared to intact tissues with altered rhythmicity (112, 113). Robustness is also ensured by interlocking-feedback loops at the genetic circuit level, for review, see Ref. (114), and has been featured in models of circadian rhythms from different organisms (17, 115–118). In mammals, genetic (119–122) and pharmacological (38, 44) perturbation of the secondary feedback loop showed that it primarily served as a stabilizing mechanism.

Modeling approaches have revealed that activator and repressor complex formation are necessary for noise resistance (123) and that a 1:1 stoichiometric balance of repressors binding activators rather than binding DNA is important for robust circadian timekeeping (124). Experiments in mammals seem to support these models because rhythm generation in mouse embryonic fibroblasts can be abolished by constitutive expression of the mammalian repressor (125) or by artificially altering the stoichiometry between activators and repressors (126). Indeed, the natural stoichiometry between activators and repressors in a mouse liver is close to 1:1 as measured by western blotting (127) and mass spectrometry (128).

The difference in repression mechanisms—Hill-type non-linearity from models based on the Goodwin oscillator or protein-based sequestration leads to subtle differences in the activity of the activator in circadian models as the concentration of the repressor increases. For Hill-type models, there is an all-or-none switch that occurs when multisite phosphorylation or cooperative binding reaches some critical level. The activator is like a light bulb that is on until it suddenly gets switched off. For protein-sequestration models, the activity of the activator linearly decreases as a function of the molar ratio between activator and repressor, which is like a light bulb slowly turned down by a dimmer. These differences can affect the synchronized period between coupled heterogeneous oscillators compared to the mean period of uncoupled oscillators (129). Importantly, understanding the differences in repression mechanisms for coupled oscillators can lead to testable predictions on how clock components interact with other proteins, such as regulation of the tumor antigen p53 (130).

## Systems Transcriptomics

### Identification of the Components of the Circadian Clock

On a tissue level, the central clock in mammals is located in a structure of the brain called the SCN. Ganglion cells in the retina detect light signals through a photopigment called melanopsin and relay this information to the SCN. SCN neurons project to different regions of the brain and synchronize biological clocks in peripheral tissues by secretion of hormones as previously reviewed (131, 132). However, most tissues in an organism have the core transcriptional architecture for circadian rhythmicity including liver, lung, and muscles (133) as well as cultured cells (134–136).

The genetic network for circadian rhythms is based on delayed feedback repression of transcription. Briefly, a CLOCK:BMAL1 heterodimer activates transcription at promoter elements called E-boxes. A protein called PERIOD (PER) heterodimerizes with another protein CRYPTOCHROME (CRY) and translocates to the nucleus where it represses transcription of the Period gene and other genes that activate Period transcription, reviewed elsewhere extensively (137–140). Several components of the core transcriptional network were identified in forward-genetics screens (i.e., random mutation of an organism’s genome and searching for mutants with abnormal rhythms) including Period (7) and Timeless (141) in *Drosophila*, Frequency (35) in *Neurospora*, and Clock (142, 143) in mice.

Systems approaches have been successful in identifying other core clock components such as Bmal, which was identified using an iterative search for other bHLH proteins (144, 145). Genomics-based strategies helped to identify activators of Bmal such as Rora (122) and Nr1d1 (121), and functional genomics strategies in *Drosophila* revealed Clockwork Orange (146–148) as a homolog of the mammalian Dec1 and Dec2 (149).

### Systems Experiments to Study the Transcriptome

Some of the earliest systems approaches to study circadian rhythms were to simply analyze all the mRNA in a tissue or organism to determine which mRNAs had cyclic expression. These studies used microarrays to identify cycling mRNAs in *Drosophila* (150, 151), in the mouse liver, heart, and SCN (152–155), rat pineal gland (156), isolated fibroblasts (157, 158), and in plants (159). There was considerable tissue specificity in rhythmic genes because only approximately 10% of cycling genes were common to at least one other tissue (160). Additionally, there are approximately 100-fold fewer cycling transcripts in NIH3T3 and U2OS cell culture models compared to mice tissue (161). This study also revealed 12-h oscillatory transcripts in liver, heart, lungs, and other tissues, but not in cultured cells (161). These “harmonic” rhythms are perturbed by a disrupted circadian clock in the SCN (162). Rhythmicity of the core clock component PER2 in these tissues could be confirmed with luminescent reporter mice (163).

Recent studies have begun to use RNA sequencing to measure steady-state mRNA expression in tissues such as the mouse liver (164–166) or to identify transcription factor-binding sites using chromatin-immunoprecipitation coupled with RNA-sequencing (CHIP-seq) (164–170). Comparative genomic approaches revealed the importance of E-boxes, D-boxes (171), and RREs (155, 171) in timing circadian mRNA expression, which have allowed ensemble-based predictions of phase response from combinations of these elements (25).

### Systems Experiments Analyzing Chromatin State

Next-generation sequencing experiments revealed both circadian initiation and recruitment of RNA polymerase II (RNAPII) to circadian promoters (164, 168) and concomitant circadian changes in chromatin state (164, 166, 168). In particular, H3K4me3 histone methylation have circadian oscillations that slightly lag RNAPII occupancy (168). Circadian regulation of chromatin state was first observed in an increase in phosphorylation of histone H3S10 in the SCN in response to light (172). Additionally, rhythmic acetylation of histone 3 was observed in the promoters of Per1, Per2, and Cry1 in mouse liver (173, 174). CLOCK itself has intrinsic histone acetylase activity (175) and is rhythmically recruited to circadian promoters (174, 176). CLOCK can acetylate other non-histone proteins including BMAL1, which promotes recruitment of CRY1 and thus BMAL1–CLOCK inactivation (177). SIRT1, a sirtuin histone deacetylase whose activity depends on the coenzyme nicotinamide adenine dinucleotide (NAD^+^), interacts with CLOCK and can deacetylate BMAL1 (174) and PER2 (178). SIRT1 also controls H3K4me3 methylation through circadian deadenylation of the histone methyltransferase mixed-lineage leukemia 1 (179). Circadian regulation results in cycles of NAD + biosynthesis (180), NAD + recycling (181), alters Clock and Bmal1 binding (182), and NAD redox rhythms have been observed directly in cells (183). Together, these studies suggest a direct link between metabolism and epigenetic regulation of circadian rhythms.

### MicroRNAs (miRNAs) in Circadian Rhythms

In addition to discovering cycling transcripts, systems transcriptomics experiments have uncovered other cycling RNAs such as long non-coding RNAs (lncRNAs) and miRNAs. For example, CHIP-seq experiments revealed clock proteins such as Clock, Bmal1, and Nr1d1 binding at sites outside of canonical gene promoters (166, 167, 169, 170, 184, 185), which suggested circadian regulation of non-protein-coding transcripts. MiRNAs bind target mRNAs typically in 3′ untranslated regions (3′ UTRs) to inhibit translation and destabilize the mRNA, for review see Ref. (186–189). Microarray studies uncovered miRNA expression inversely correlated with circadian activators Clock and Bmal1 and positively correlated with circadian suppressors Per, Cry1, and Nr1d1 (190), and other miRNAs that have diurnal expression patterns (191). MiRNAs are regulated by circadian proteins such as CLOCK (170, 192, 193) and NR1D1 (194) and modulate the expression of circadian genes such as Bmal1 (195–198), Clock (193, 199, 200), the circadian polyA deadenylase Nocturnin (201), Per1 and Per2 (202–204), Clockwork Orange (205), Timeless in *Drosophila* (206), and Cry1 (207). Knockout of the core miRNA-processing machinery in mouse liver revealed that ~30% of the rhythmic transcriptome is posttranscriptionally modulated by miRNAs (208).

### lncRNAs in Circadian Rhythms

In addition to miRNAs, next-generation sequencing experiments have revealed extensive transcription of lncRNAs (209, 210) and circadian expression of lncRNAs (166, 211, 212). An in depth study revealed differential expression of 112 lncRNAs in the rat pineal gland, and light expression at night could modulate the level of some of these lncRNAs (213). A study of mouse liver revealed 19 out of 123 lncRNAs detected with robust oscillations and detected antisense transcripts associated with Per2 (166). Antisense transcription of Per2 in mice liver has been reported by others (164, 165) and originally in the silk moth (214), but it remains unclear what the function of antisense Per2 is for circadian rhythms. In *Neurospora*, the antisense transcript of frequency (called Qrf—Frq, spelled backward) is important for entrainment to light, oscillates in a reciprocal pattern to Frq, and promotes Frq gene silencing via heterochromatin formation (215–217). Deletion of a lncRNA associated with Prader–Willi syndrome in mice results in increased energy expenditure and altered expression of circadian genes such as Clock, Cry, and Per (218). Additionally, a lncRNA highly upregulated in liver perturbs the expression levels of Clock, Cry, and Per in hepatoma cells (219). Together, these studies suggest a role for non-protein-coding transcripts in the regulation of circadian rhythms.

### Posttranscriptional Regulation of Circadian Rhythms

Next-generation sequencing studies have also examined to what extent rhythmic steady-state mRNA transcripts result from *de novo* rhythmic transcription versus rhythms via posttranscriptional regulation. By analyzing expression of introns as an indicator of pre-mRNA levels, a study by Koike et al. determined that the majority circadian mRNAs do not undergo rhythmic transcription (164). Another method to directly assess *de novo* transcription called Nascent-seq confirmed this result and further showed that many mRNAs with *de novo* rhythmic transcription do not have rhythms in steady levels of mRNA (165). A similar nascent-seq study in *Drosophila* also revealed a considerable posttranscriptional contribution to cycling mRNA amplitudes (220).

There are a variety of mechanisms for posttranscriptional regulation of circadian rhythms including splicing, mRNA export, polyadenylation, mRNA stability, methylation, and regulated translation—for review, see Ref. (221). The first indication of posttranscriptional regulation of circadian rhythms was that stability of *Drosophila* Per mRNA oscillates (222), which was also later observed in mammals (223). Posttranscriptional regulators such as LARK bind to the 3′ UTR of Per1 mRNA to enhance PER1 translation (224, 225). LARK also promotes alternative translation of the casein kinase homolog Doubletime in *Drosophila* (226). Researchers have uncovered other proteins that regulate translation of clock components. For example, the heterogenous nuclear ribonucleoprotein Q (hnRNP Q) modulates translation of Nr1d1, Per1, Per3, Cry1, and the rate-limiting enzyme in melatonin synthesis AANAT (227–232). Cry1 mRNA stability is also regulated by AU-rich element RNA-binding protein (AUF1) also known as hnRNP D (233, 234), and Per2 mRNA stability was found to be modulated by polypyrimidine tract-binding protein also known as hnRNP I (235).

### mRNA PolyA Tail Length and Circadian Rhythms

Other mRNA processing mechanims may also posttranscriptionally regulate circadian rhythms. The 3′ end of newly transcribed pre-mRNA in the nucleus is cleaved and a polyA tail is added at one of the several possible sites (236). Deadenylation of this polyA tail in the cytoplasm by enzymes such as the poly(A)-specific ribonuclease and the Ccr4-Not complex can shorten tail length and accelerate mRNA degradation (237, 238). Daily variation in polyA tail length was first observed for vasopressin mRNA in the SCN (239). In *Xenopus*, another deadenylase called Nocturnin was discovered in a screen to detect rhythmically expressed mRNAs in retinal photoreceptors (240, 241) and was later shown to be expressed in multiple mouse tissues (242). Nocturnin is one of the few mRNAs that remain rhythmic after the liver clock is conditionally inactivated by drug-mediated Bmal1 expression (243) and can be posttranscriptionally regulated by miR-122 (201). Mice lacking Nocturnin do not have any obvious circadian behavior deficiencies, but are resistant to diet-induced obesity (244). However, in *Drosophila*, loss of Nocturnin results in abnormal behavior rhythms in constant light (245). A microarray method to measure polyA tail length suggested that rhythmic nuclear adenylation is coupled to rhythmic transcription and that rhythmicity in polyA tail length is related to rhythmic protein expression (246). These studies suggest that posttranscriptional regulation by deadenylation may be important for proper circadian rhythms and that next-generation sequencing techniques such as polyA tail profiling (247, 248) will be critical for fully understanding the contribution of polyA tail length to circadian rhythms.

### Systems Experiments to Measure mRNA Modification, Structure, and RNA-Binding Proteins

Besides polyadenylation, mRNA processing by other mechanisms may contribute to circadian rhythms. A recent study showed that reduction of Mettl3, an m^6^A mRNA methylase involved in mRNA processing and nuclear export, reduces m^6^A methylation of circadian transcripts and extends period (249). Next-generation sequencing studies of m^6^A methylation may reveal other contexts in which methylation of mRNA is important for circadian rhythms (250). In addition, other RNA-sequencing techniques to probe RNA secondary structure such as dimethyl sulfate sequencing—DMS-seq and parallel analysis of RNA structure—PARS-seq (251, 252), BRIC-seq for mRNA stability (253, 254), and various methods to analyze RNA-binding sites of specific RNA-binding proteins such as CLIP, CLIP-seq, HITS-CLIP, iCLIP, and PAR-CLIP (255–260) will be critical for understanding how mRNA processing is involved in circadian rhythms. For example, CLIP-seq of mRNAs bound to cold-inducible binding protein, which is required for high-amplitude circadian gene expression, revealed binding to Clock and other circadian transcripts (261).

## Systems Proteomics and Metabolomics

### Circadian Proteomics

Researchers are beginning to use systems approaches to study the circadian proteome and metabolome. Using two-dimensional difference gel electrophoresis (2D-DIGE), Reddy and colleagues revealed that approximately 20% of the soluble proteins in the mouse liver oscillate. Surprisingly, for many rhythmic proteins, the corresponding mRNA was not rhythmic, which suggests translational and posttranslational control of protein rhythms (262). 2D-DIGE has also been used to investigate circadian differences in the mouse retina (263) and day and night differences in the mouse heart (264). In addition to mice, 2D gel-based mass spectrometry has been used to investigate chronological changes in eukaryotic algae (265, 266) and in plants (267, 268).

Other groups have employed stable isotope labeling by amino acids in cell culture (SILAC) to compare two groups of samples—one mixed with “heavy” amino acids and one mixed with “light” amino acids based on the composition of different element isotopes (269). SILAC-based quantitative mass spectrometry has been used to uncover cycling proteins in the mouse liver (270, 271) and SCN (272). Traditional SILAC approaches use chemical synthesis of peptides with isotopically labeled amino acids (269, 273) or gene expression systems in *E. coli* (274, 275). However, cell-free protein synthesis systems are potentially a more cost-effect tool to express isotope-labeled peptides because the volume of the reaction is much lower and purification is easier because there is no need for culturing, harvesting, and disrupting cells (275–278). Recently, a cell-free protein synthesis system called the PURE system (279) coupled with high-resolution mass spectrometry in a workflow called MS-QBiC was used to quantify 20 selected circadian clock proteins over a 24-h time series (128). This study estimated the absolute number of protein molecules for core clock components per cell and the delay between steady-state levels of mRNA (measured by qPCR) and protein copy number (128).

In addition to SILAC, label-free approaches such as MaxLFQ (280) have been used to quantify proteins in mouse skeletal muscle (281). Mass spectrometry has been used to examine the global proteome in cyanobacteria (282). Mass spectrometry has also been used to analyze the global phosphoproteome and revealed ~5,000 phosphosites that significantly oscillate in the mouse liver (283) and ~3,000 phosphosites in *Arabidopsis* (284). Given the widespread discrepancies between transcript and protein rhythmicity in a number of organisms, in the future, it will be useful to understand the role of translation and posttranslational regulation as well as cycling protein modification states (e.g., phosphorylation) to circadian networks.

### Circadian Metabolomics

Researchers have looked at rhythmic metabolites in humans (285–288) and in mice (289–292) and have shown that circadian proteins directly regulate metabolism (44, 184). Researchers have also used comprehensive metabolite profiling to analyze diet effects in mice (293–295) and the effects of sleep loss in humans (296–299). Computational databases have been developed to compare published transcriptomes, proteomes, and metabolomes (292). Metabolic profiling is still quite noisy compared to transcriptome data at least for identifying tissue-specific signatures (300), and many challenges remain including identification of unknown metabolites, standardization of data repositories and reporting methods, and integration with other types of data. Researchers are beginning to use metabolic profiling over larger time courses and with higher resolution in cell culture lines (301). In the future, coupling these methods with gene knockout or knockdown of core clock components will enable researchers to identify connections between circadian rhythms and metabolism. For example, are there harmonics in metabolite rhythms (i.e., multiples of a 24-h rhythm like 8- and 12-h rhythms) similar to the harmonics of mRNA rhythms (161, 162), and would these rhythms be influenced by circadian genes?

One benefit of systems studies is the development of a molecular timetable to detect an individual’s body time based on a single time point assay. Molecular timetables have been developed with mice transcriptome data (212, 302) and applied to mice (291) and human (288) metabolite data, proteomic data (128), and even human breath (303). In theory, metabolite timetables could enable researchers to hone chronotherapeutic strategies for clinical conditions. However, despite the strong evidence that circadian timing effects xenobiotic metabolism, bioavailability, and drug efficacy and that many of the most successful drugs in the United States target proteins with circadian rhythm components (212), ongoing clinical trials rarely exploit time-of-day-dependent drug delivery (304).

## Systems Approaches to Study Translation Regulation in Circadian Rhythms

Although 10% of genes are rhythmic in the liver (152), *de novo* transcription is only responsible for a small fraction of this rhythmicity (164). Thus, gene expression studies using microarrays and RNA-sequencing may not correlate with translation of the corresponding mRNA nor with protein abundance (305). In the mouse liver, systems studies of the proteome are unable to detect low-abundant components of the core circadian circuit (270, 271), unless special care is taken to examine a particular protein on a case-by-case basis (128). Thus, researchers have begun to use next-generation sequencing techniques of mRNA attached to mRNA in monosomes and polysomes (306, 307) and with affinity purification (308–310) as a proxy for protein abundance and to understand how translation regulation affects protein abundance.

It has been known for more than 50 years that perturbation of translation disrupts circadian rhythms (311). Until recently, there has been a shortage of good tools to measure translation directly. In 2009, Nicholas Ingolia in Jonathan Weissman’s lab developed a technique called ribosomal profiling, which uses RNA sequencing of ribosome-bound mRNA protected from RNAse degradation, to determine the location and abundance of ribosomes in the yeast transcriptome (312). Researchers have begun to use this method to study circadian rhythms in ribosomal occupancy (313, 314). These studies discovered a class of rhthmically translated mRNAs without corresponding steady-state mRNA rhythms (313, 314), which in the case of mouse liver may be a result of rhythmic ribosomal biogenesis (315). Researchers have previously observed that global translation is rhythmic in the mouse liver (316, 317), which is probably a result of activation of the TORC1 pathway (315, 318–320). Interestingly, diurnally regulated translation in the mouse liver is only moderately affected by knockout of the core clock component Bmal1 and many genes that contained 5′-terminal oligo pyrimidine tract or translation initiator of short 5′ untranslated region (5′-UTR) sequence have rhythms in ribosomal occupancy independent of trancriptional rhythms (321). These studies in addition to previous research (322–326) suggest that feeding rhythms can synchronize the liver in the absence of cues from neuronal pacemaker cells in the SCN.

The Janich and Jang studies (313, 314) also revealed widespread circadian translation of upstream open reading frames (uORFs) in 5′ UTRs. Translation of uORFs globally represses translation efficiency—a measure of the ratio of ribosomal occupancy, determined by ribosomal profiling, to steady-state mRNA, measured by RNA-sequencing (314). Interesting, many circadian mRNAs also have uORFs in their 5′ UTRs (Table 1), which may disrupt translation of the downstream coding sequence by ribosomal pausing on the mRNA, alternative translation, or other mechanisms (327). Ribosome pausing on uORFs may be alleviated by the action of the non-canonical initiation factors density regulated protein (DENR) and multiple copies in T-cell lymphoma (MCT-1), which act to promote translation reinitiation downstream of uORFs (328, 329). Depletion of DENR by shRNAs in NIH3T3 cells shortens the period by 1.5 h, which suggests that uORFs may be relevant for circadian function (314). In other biological contexts, repression of translation by uORFs can be regulated by trans-acting factors. For example, in *Drosphila*, the master switch gene Sex-lethal (Sxl) is important for sex, for review see Ref. (330–332). SXL-binding downstream of a short uORF on *male-specific lethal (msl)-2* enhances translation repression by the uORF on downstream reading frame translation (333). During mitosis, one of the most translationally repressed mRNAs is early mitotic inhibitor 1 (Emi1) that inhibits the activity of the anaphase-promoting complex (334). Emi1 has multiple transcript isoforms and the isoform with several uORFs in the 5′ UTR is severely crippled for translation initiation in single-molecule reporter experiments (335). These studies suggest that uORF-mediated translational repression is important in a variety of biological functions and may have an unexplored role in circadian rhythms.

**Table 1:** **Number of upstream open reading frames (uORFs) in common circadian clock genes**.

Gene name	Ref Seq ID	Number of uORFs	uORF length (nt)
Bhlhe40	NM_011498	1	18

Bmal1	NM_007489 NM_001243048	4 2	72; 42; 21; 33 201; 171

Clock	NM_007715 NM_001289826	3 4	66; 48; 30 339; 66; 48; 30

Cry1	NM_007771	2	36; 24

Cry2	NM_009963	0	—

CK1d	NM_139059	2	27; 21

CK1e	NM_013767 NM_001289898 NM_001289899	0 0 2	— — 126; 66

Dbp	NM_016974	2	12; 42

Nfil3	NM_017373	3	15; 51; 12

Nr1d1	NM_145434	3	117; 192; 21

Nr1d2	NM_011584	3	120; 120; 117

Per1	NM_011065 NM_001159367	1 1	15 15

Per2	NM_011066	1	6

Per3	NM_011067 NM_001289877 NM_001289878	4 4 4	63; 30; 84; 48 63; 30; 84; 48 63; 30; 84; 48

Rorc	NM_011281	0	—

Tef	NM_017376 NM_153484	1 0	291 —

What is the consequence of disrupted translation for circadian rhythms? One clue came when researchers showed that codon usage affects circadian function in cyanobacteria (336), *Neurospora* (337), and *Drosophila* (338). While cyanobacteria with codon-optimized Kai genes have enhanced circadian rhythmicty at cooler temperatures, this modification impairs cell growth, which suggests that non-optimal translation could provide an adaptive response to changes in the environment (336). In *Neurospora*, codon optimization of Frq alters FRQ protein structure, which impairs circadian rhythms (337). Similarly, in *Drosophila*, codon optimization results in conformational changes of the *Drosophila* PER protein altering PER phosphorylation, stability, and impairs behavioral rhythms (338). Additionally, it is becoming clear that translation control is interlinked with both circadian rhythms and sleep disorders. For example, Ataxin2 functions as a critical translation activator of Per2 in flies (339, 340), and individuals with disease mutations in human Ataxin2 have disturbed rapid eye movement sleep (341, 342).

## Systems Neurophysiology

Systems neurophysiologists are beginning to connect the circadian circuit to more complex outputs from the clock such as activity rhythms. Forward genetics in mice have already uncovered core components in the circadian network (142), and researchers have begun to use forward genetics for complex behavior such as sleep (343). On the other hand, the development of TALEN (344), Zinc-Finger Nucleases (345), and CRISPR/Cas9 (346, 347) gene knockout systems have accelerated the pace at which researchers can pursue reverse genetics in mice. In particular, CRISPR/Cas9 systems have been extensively modified to improve targeting efficiency and specificity (346–353). However, the need for invasive techniques such as electroencephalography and electromyography to characterize sleep hampers high-throughput phenotyping. To facilitate rapid phenotyping, researchers have developed a respiration-based, sleep staging system in combination with redundant CRISPR targeting to reveal new genes important for sleep regulation (354, 355). In particular, researchers generated and analyzed more than 21 different KO mice and discovered different ion channels that could increase or decrease sleep duration (355). These studies have revealed the genetic bases for behaviors such as sleep, but do not show how neural networks and structures in the brain are wired to carry out such behavior. In the past, researchers have used conventional histology and immunohistochemistry of sliced brain sections to reveal the when and where of gene function, but recent advances in tissue clearing have begun to enable direct imaging of intact organs (356).

Optical sectioning using light-sheet microscopy in combination with recently developed tissue-clearing techniques is a potent strategy to begin to explore the neuroanatomical basis of behavior (357–362). Image analysis algorithms, automated comparative analysis, and feature extraction will enable researchers to quickly test and analyze neural activity in different parts of the brain with different mutant mice and under a variety of experimental conditions. These approaches will be useful to determine what areas of the brain are affected by sleep/wake pharmaceutical reagents such as methamphetamine and to develop a whole-brain anatomical atlas to catalog and characterize every individual cell in the brain.

## Conclusion

Systems experiments from modeling to metabolomics have significantly increased our understanding of circadian rhythms, but many challenges remain. For modeling, we still do not have a comprehensive understanding of temperature compensation nor the role individual enzymes have in temperature-independent and -dependent reactions. We do not understand the contribution of temperature-compensating reactions at the molecular, cell, tissue or organism level and how these temperature-compensating systems interface with one another. At an enzymatic level, we could learn much by designing and building *de novo* temperature-compensated reactions or by converting temperature-sensitive enzymes into temperature-compensating ones. There is also a need for understanding how recently discovered posttranslational oscillators such as the peroxiredoxin system interface with the genetic circadian circuit, and for an evolutionary investigation into how and why these distinct circadian timekeeping systems arose. Modeling is needed to make connections between different timekeeping systems, different organization levels of timekeeping from molecule to tissue, and between circadian rhythms and other rhythms such as the cell cycle.

For systems “–omics” researchers, there is a large variation in the rhythmicity of transcripts, metabolites, and proteomes detected even with similarly defined experimental systems. This may be in part due to how different algorithms detect rhythmicity (153, 363, 364), differences in sampling intervals (every 2, 3, 4, or 6 h), sampling duration, environmental conditions, and biological variability (365). As surveys of the circadian proteome increase, there has been an increasing realization of the widespread gap between transcript rhythms and protein levels. Posttranscriptional and posttranslational studies that examine mRNA structure and processing, translation, and protein modification will enhance our understanding of how transcriptional rhythms become protein rhythms, and how rhythms could evolve without genetic underpinnings.

For systems neurophysiologists, there is a pressing need to develop fast and reproducible assays that connect behavioral phenotypes to particular features and neurons in the brain and other tissues. Developments in computational processing power, data storage, and deep learning approaches will aid researchers in handling and analyzing the overwhelming amount of data generated by systems studies. Nevertheless, it will be important to validate findings with molecular techniques, case studies, and synthetic biology approaches to reconstitute behavior. Finally, can we translate this knowledge base to relevance in the clinic? It will be important to develop new assays and algorithms for body time estimation from samples at one or two time points. A combination of transcriptome, metabolome, and proteome timetables may further reduce the need for additional samples and increase accuracy of body time estimation. Integration of chronotherapeutics to clinical trial design and dosing protocols may enhance the success of drug candidates and perhaps lead to a reevaluation of the timing of drug delivery to achieve the greatest benefit to patients.

## Author Contributions

Discussed and conceived of the direction of the review: AM and HU. Illustrated the table and figure and wrote the paper: AM.

## Conflict of Interest Statement

The authors declare that the research was conducted in the absence of any commercial or financial relationships that could be construed as a potential conflict of interest.
